# The effect of preprocessing filters on predictive performance in radiomics

**DOI:** 10.1186/s41747-022-00294-w

**Published:** 2022-09-01

**Authors:** Aydin Demircioğlu

**Affiliations:** Institute of Diagnostic and Interventional Radiology and Neuroradiology, University Hospital Essen, Hufelandstraße 55, 45147 Essen, Germany

**Keywords:** Artificial intelligence, Benchmarking, Machine learning, Precision medicine, Radiomics

## Abstract

**Background:**

Radiomics is a noninvasive method using machine learning to support personalised medicine. Preprocessing filters such as wavelet and Laplacian-of-Gaussian filters are commonly used being thought to increase predictive performance. However, the use of preprocessing filters increases the number of features by up to an order of magnitude and can produce many correlated features. Both substantially increase the dataset complexity, which in turn makes modeling with machine learning techniques more challenging, possibly leading to poorer performance. We investigated the impact of these filters on predictive performance.

**Methods:**

Using seven publicly available radiomic datasets, we measured the impact of adding features preprocessed with eight different preprocessing filters to the unprocessed features on the predictive performance of radiomic models. Modeling was performed using five feature selection methods and five classifiers, while predictive performance was measured using area-under-the-curve at receiver operating characteristics analysis (AUC-ROC) with nested, stratified 10-fold cross-validation.

**Results:**

Significant improvements of up to 0.08 in AUC-ROC were observed when all image preprocessing filters were applied compared to using only the original features (up to *p* = 0.024). Decreases of -0.04 and -0.10 were observed on some data sets, but these were not statistically significant (*p* > 0.179). Tuning of the image preprocessing filters did not result in decreases in AUC-ROC but further improved results by up to 0.1; however, these improvements were not statistically significant (*p* > 0.086) except for one data set (*p* = 0.023).

**Conclusions:**

Preprocessing filters can have a significant impact on the predictive performance and should be used in radiomic studies.

**Supplementary Information:**

The online version contains supplementary material available at 10.1186/s41747-022-00294-w.

## Key points

- Modeling using all preprocessed features did not reduce the predictive performance compared to the original (not preprocessed) set.
- Tuning image processing filters during cross-validation improves the performance slightly.
- Using all preprocessed features seemed to be more helpful for datasets with larger sample size.
- Pairwise Pearson correlations of preprocessed feature sets were high (*r* > 0.49).

## Background

Radiomics characterises pathologies in medical images using quantitative features to enable better, personalised, and noninvasive diagnosis [1]. It exploits on machine learning pipelines that can be traced back to the 1970s [2], where a large set of quantitative features is first extracted from the image data and then used to train an appropriate machine learning model. Since features are critical for higher predictive performance, their expressiveness is often increased by applying preprocessing filters to the image data before feature extraction. Examples of such preprocessing filters are smoothing filters, which can reduce the image noise, exponential filters, which can highlight subtle differences in the data, and wavelet filters, which take into account the spectral dimension of the data [3].

However, the use of preprocessing filters can easily increase the number of extracted features from a few dozen to several thousand while the number of samples remains constant. Combined with the small sample sizes that are often the case in radiological studies, this leads to high-dimensional datasets characterised by containing more features than samples. Such datasets are notoriously complex and produce relatively unstable and sometimes spurious results [4]. In addition, some features may not change significantly due to preprocessing, leading to a higher correlation in the datasets. Intuitively, adding more features to a problem should not decrease predictive performance if the new features contribute to the solution. However, if they do not contribute to the solution, the model could simply disregard the added features; the performance would remain the same. Regrettably, this is only true in theory because, in practice, the sample sizes are often very small, and the features are noisy. Furthermore, given the high dimensionality of the data, features may correlate with the target by chance and, therefore, would not be disregarded during training, leading to a degradation in predictive performance.

While several studies have already been conducted to understand the influence of preprocessing filters on the variability and reproducibility of preprocessing filters [5–8], their impact on the predictive performance of subsequent models has not yet been considered. Consequently, it is unclear whether the added complexity of using preprocessing filters significantly increases prediction performance or whether the increased dimensionality negates the intended benefit. In this study, therefore, we investigated the extent to which the use of preprocessed features affects the prediction performance on several datasets.

## Methods

### Ethical statement

In this study, only previously published, publicly available datasets were used. The corresponding ethical review boards have already granted approvals for each dataset. Ethical approval for this study was waived by the local Ethics Committee (Ethik-Kommission, Medizinische Fakultät der Universität Duisburg-Essen, Germany). The study was performed following the relevant guidelines and regulations.

### Datasets

We used seven publicly available datasets (Table 1); six were published as the “WORC” database [9], while one, the head & neck cancer dataset, was published in the seminal paper by Aerts et al. [10]. Datasets were retrieved by using the Python code in the “WORC” dataset. Upon inspection, a few scans have been removed from the datasets since their slice thicknesses were larger than twice the median slice thickness of the other scans; in addition, two scans were not readable and were removed as well. These problems mainly affected the Desmoid and Melanoma datasets, where 8 and 6 scans were removed, respevtively (Table 1). All datasets contained segmentations of the corresponding pathology.

**Table 1:** Datasets used in the experiments

Dataset	Modality	***N*** (excluded)	In-plane resolution (mm)	Slice thickness (m)
CRLM	CT	76 (1)	0.7 (0.6–0.9)	5.0 (1.0–8.0)
Desmoid	MRI	195 (8)	0.7 (0.2–1.8)	5.0 (1.0–10.0)
GIST	CT	244 (2)	0.8 (0.6–1.0)	3.0 (0.6–6.0)
HN	CT	134 (1)	1.0 (1.0–1.1)	3.0 (1.5–3.0)
Lipo	MRI	113 (2)	0.7 (0.2–1.4)	5.5 (1.0–9.1)
Liver	MRI	186 (0)	0.8 (0.6–1.6)	7.7 (1.0–11.0)
Melanoma	CT	97 (6)	0.7 (0.5–1.0)	1.2 (0.6–2.0)

*N* denotes the number of samples, with the number of scans removed from the original dataset in parenthesis (excluded); for example, there were 203 scans in the Desmoid dataset, 8 of which have been removed. In-plane resolution and slice thickness are reported as median (range). *CT* Computed tomography, *MRI* Magnetic resonance imaging. For the datasets, see references [9, 10]

### Image preprocessing

All scans, including the segmentations, were resampled using spline interpolation to a homogenous voxel size of 1 mm^3^. MRI series were intensity normalised before processing by centring all values to have a mean of 0 and a standard deviation of 1. CT series were not normalised since HU values were comparable between scans.

### Preprocessing filters

Before feature extraction, the following filters were applied to the scans: exponential; gradient; Laplacian-of-Gaussian (LoG); local binary pattern in two- and three-dimensions; logarithm; square; square-root; and wavelet. More details on the parameters can be found in Table 2.

**Table 2:** Overview of the image filters

Filter	Parameters	***N***
Original	–	105
Exponential	–	91 (196)
Gradient	–	91 (196)
Laplacian-of-Gaussian	Sigma 1.0, 2.0, 3.0, 4.0, 5.0 mm	455 (560)
Local binary pattern	Two-dimensional, three-dimensional	273 (378)
Logarithm	–	91 (196)
Square	–	91 (196)
Square root	–	91 (196)
Wavelet	Directions HHH, HHL, HLH, HLL, LHH, LHL, LLH, LLL	728 (833)

*Original* denotes the set of extracted features without any image processing, which also contains of shape features. *Parameters* denote the chosen extraction parameters for the preprocessing filter, if applicable. HHH, HHL etc. denote the frequency components of the wavelet filters (H = high, L = low). *N* denotes the number of features that were generated after applying the filter; in parenthesis is the sum of the number of original features and the features after applying the corresponding filter

### Feature extraction

The width of discretisation bins for feature extraction was fixed to 25. The standard feature classes were extracted: shape; first order; grey level co-occurrence matrix, GLCM; grey level size zone matrix, GLSZM; grey level run length matrix, GLRLM; neighboring grey-tone difference matrix, NGTDM; and grey level dependence matrix, GLDM. Altogether, 2016 features were generated for each dataset. The complete list of features is provided in the Supplemental material. PyRadiomics 3.0.1 [11] was used for extraction. Details can be found in the software package and the source code.

### Feature sets

**Fig. 1:**
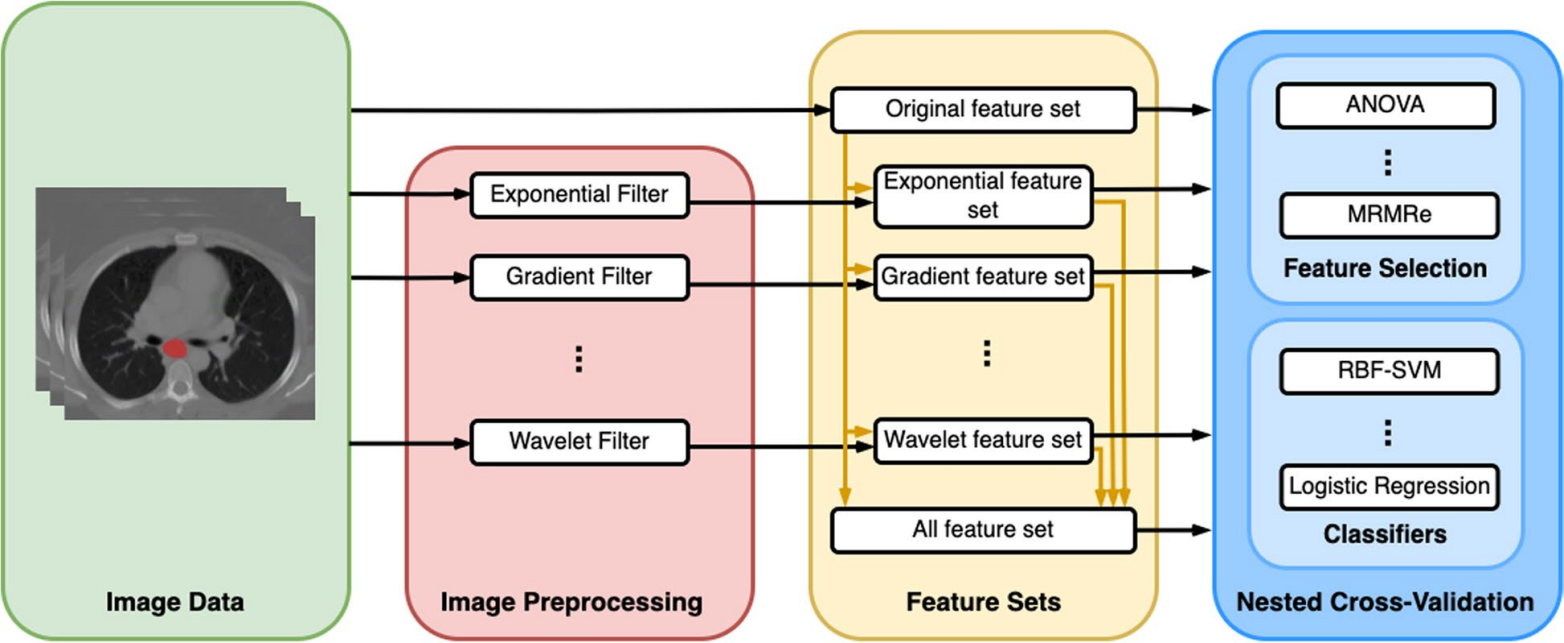
Overall pipeline used for training. The image data was preprocessed by each filter to generate the different feature sets. A 10-fold nested, stratified cross-validation was applied to train a predictive model

Different feature sets were created (Fig. 1): The original features, extracted from the images with no preprocessing filter applied, formed the baseline. Then, a distinct feature set was computed for each preprocessing filter to yield a feature set specific to the filter. The original features were added to each feature set since the original features contained morphological features like volume, which are known to have high predictive value. Finally, all features were merged to form a single-feature set containing all features. Altogether, ten feature sets were created.

### Feature preprocessing

Before training, all extracted features were normalised using column-wise *z*-scores. No missing values were present. Constant features were removed from the datasets.

### Feature selection methods

For the selection of relevant features, five often used feature selection algorithms were used: analysis of variance, ANOVA; Bhattacharyya distance; extra trees; least absolute shrinkage and selection operator (LASSO); and minimum redundancy maximum relevance ensemble, mRMRe. In addition, no feature selection method was applied, *i.e.,* the classifiers processed all features. The LASSO algorithm had a hyperparameter C, which was fixed at 1.0. Because these algorithms except for the LASSO are not selecting but scoring each feature, a choice had to be made on how many of the highest-scoring features should be used for classification. This choice was treated as a hyperparameter and was chosen among 1, 2, 4, 8, 16, 32, and 64. For the LASSO, the absolute values of the selected coefficients were used as a proxy for the scores.

### Classifiers

The following five algorithms were used: logistic regression; naive Bayes; neural networks, random forests; and support vector machine with a non-linear kernel (radial basis function sector vector machine). Apart from naive Bayes, which can be considered a baseline, all methods have hyperparameters that were tuned during training. More details can be found in the Supplemental material.

### Training

Models were created following the standard radiomics pipeline (Fig. 1) [12]. The training was performed for each combination of the feature selection method, classifier, and corresponding hyperparameters (Table 3) using nested 10-fold stratified cross-validation [13]. The data was divided into ten equally sized folds and processed in rounds. In each round, one fold was left out for testing, while on the other nine folds, an inner stratified 10-fold cross-validation was performed to determine the best-performing (or tuned) model. This model was then retrained and evaluated on the left-out fold. All predictions of the outer cross-validation were then pooled to obtain a single receiver operating characteristics (ROC) curve, *i.e.,* microaveraging was applied.

**Table 3:** List of hyperparameters tuned during cross-validation

Classifier	Hyperparameter
Logistic regression	C in 2^{-6, -4, -2, 0, 2, 4, 6}
Neural networks	Three layers with each 4, 16, or 64 neurons
Random forest	Number of estimators 50, 125, or 250
Radial basis function Sector vector machine	C in 2^{-6, -4, -2, 0, 2, 4, 6}, gamma was set to auto

The remaining hyperparameters were left at their default

### Evaluation

The evaluation focused on whether the feature sets differed in their predictive performance as measured by area-under-the-curve (AUC) at ROC analysis (AUC-ROC). Of central interest was the difference between three feature sets: The original feature set, the set of all features, and the best-performing feature set, as determined during internal cross-validation. In addition, the tradeoff between sample size and the difference in the predictive performance of these feature sets was examined using linear regression to detect if an association was present or not. Finally, pairwise Pearson correlations were computed to understand how the feature sets were correlated.

### Software

Python 3.6 was used for all experiments. Feature selection methods and classifiers were mainly employed from scikit-learn 0.24.2 [14].

### Statistics

Descriptive statistics were reported as mean ± standard deviation. ROC curves were compared using DeLong tests. The *p* values below 0.05 were considered to be statistically significant. No correction for multiple testing was applied due to the small sample sizes and the explorative nature of this study. Statistics were computed using Python 3.6 with the scipy module and R 3.4.1 with the pROC library.

## Results

A total of 16,570 = (6 × 6 × 10 × 46) + 10 models were fitted per dataset, each with 10-fold cross-validation, since 6 different feature selection methods (including None, which selects all features), 6 different numbers of selected features, 10 different feature sets, and 46 different classifier hyperparameter configurations were used, and the best 10 configurations were retrained for each dataset.

### Computation times

Compared to the original feature set, computation times, measured as the time required for feature selection and classification, increased on average by 1.45 times when the complete set of image preprocessing filters was computed (12.9 *versus *18.7h). On the other hand, tuning resulted in an even higher increase in computation times (138.3h). Compared to the original feature set, an increase of 10.72 times was observed, whereas 7.40 times was observed compared to the full set of features.

### Predictive performance

**Fig. 2:**
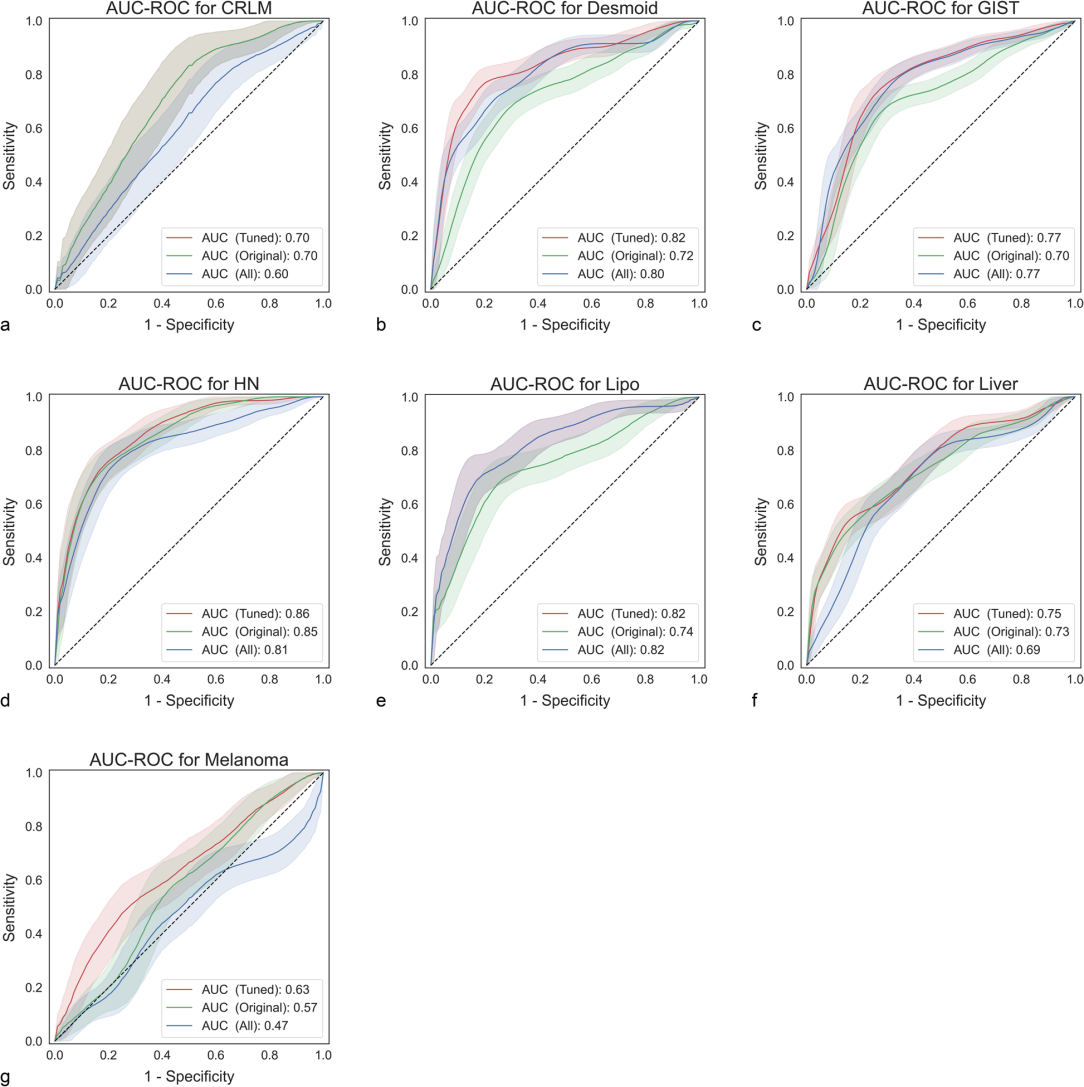
Receiver operating characteristic curves for all datasets. Tuned denotes the best-performing preprocessing filter and can differ for each dataset. Note that in the case of CRLM dataset, the best preprocessing filter set was the original set; therefore, these curves coincide

The ROC-AUCs ranged from 0.57 to 0.85 for the original feature set and 0.53 to 0.82 for all features. Using the best performing feature set identified in the cross-validation resulted in ROC-AUCs between 0.63 and 0.86 (Table 4). The original set of features performed best for CRLM dataset only. Using all features performed best in two cases, while filter tuning achieved the highest ROC-AUCs in four datasets (Fig. 2).

**Table 4:** Areas-under-the-curve at receiver operating characteristics of all the models

Dataset	AUC-ROC Original	AUC-ROC All	AUC-ROC Tuned	Δ_All—Original_	p_All—Original_	Δ_Tuned—Original_	p_Tuned—Original_	Δ_Tuned—All_	*p*_Tuned—All_	Best filter
CRLM	**0.7** (0.58–0.82)	0.6 (0.47–0.73)	0.7 (0.58–0.82)	-0.1	0.237	0	1	0.1	0.237	Original
Desmoid	0.72 (0.64–0.79)	0.8 (0.73–0.87)	**0.82** (0.76–0.89)	0.08	**0.024**	0.1	**0.006**	0.02	0.261	Wavelet
GIST	0.7 (0.63–0.77)	**0.77** (0.71–0.83)	0.77 (0.71–0.83)	0.07	**0.031**	0.07	**0.031**	0	1	All
HN	0.85 (0.79–0.92)	0.81 (0.74–0.89)	**0.86** (0.8–0.92)	-0.04	0.216	0.01	0.651	0.05	0.086	Square root
Lipo	0.74 (0.65–0.83)	**0.82** (0.74–0.9)	0.82 (0.74–0.9)	0.08	**0.04**	0.08	**0.04**	0	1	All
Liver	0.73 (0.65–0.8)	0.69 (0.61–0.77)	**0.75** (0.67–0.82)	-0.04	0.393	0.02	0.573	0.06	0.188	Square root
Melanoma	0.57 (0.45–0.68)	0.53 (0.41–0.64)	**0.63** (0.52–0.74)	-0.04	0.179	0.06	0.322	0.1	**0.023**	Exponential

The best-performing model in terms of area-under-the-curve at receiver operating characteristics (AUC-ROC) is denoted by bold face. **Δ** denotes the difference between the models; for example, a positive **Δ**_**All—Original**_ denotes an improvement using all features over the original feature set. The reported *p* values correspond to a DeLong test between the denoted models. Statistically significance (*p* < 0.05) is also denoted by bold face

Although in four datasets, the original feature set performed better than using all features, in these cases, the difference was not statistically significant, with *p* values ranging from 0.179 to 0.393. However, for the remaining three data sets where the use of all features performed better, the difference was significant. Altogether, the original feature set never performed statistically significantly better than using all features.

Furthermore, the ROC-AUCs obtained using the best-performing feature set was never worse than those of the original feature set. However, on four datasets, the difference was statistically not significant, with *p* values ranging from 0.322 to 1.0. A similar picture arises when comparing all features with the best feature set: no drop in AUC was observed, and there was a statistically significant difference in only one case (melanoma).

Regarding the best performing filter, only the wavelet, square root, and exponential filter were best; the other filters did not perform better (Table 4).

### Tradeoff against sample size

**Fig. 3:**
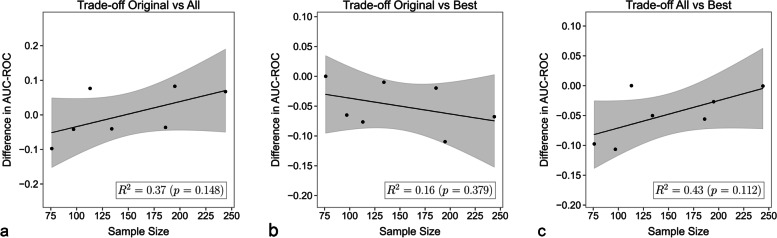
Tradeoffs against sample size. Graphical display of the association of the difference in area-under-the-curve at receiver operating characteristics between the three feature sets and the sample size. Each point corresponds to one of the datasets. **a** Tradeoff between the original set and all features. **b** Tradeoff between the original set the best-performing feature set. **c** Tradeoff of all features and the best-performing feature set

The association of the differences in the predictive performance of the three feature sets (original, all, tuned) to the sample size was considered for each dataset. However, no statistically significant increase or decrease was observed, with *p* values ranging from 0.112 to 0.379 (Fig. 3).

### Correlation of features

**Fig. 4:**
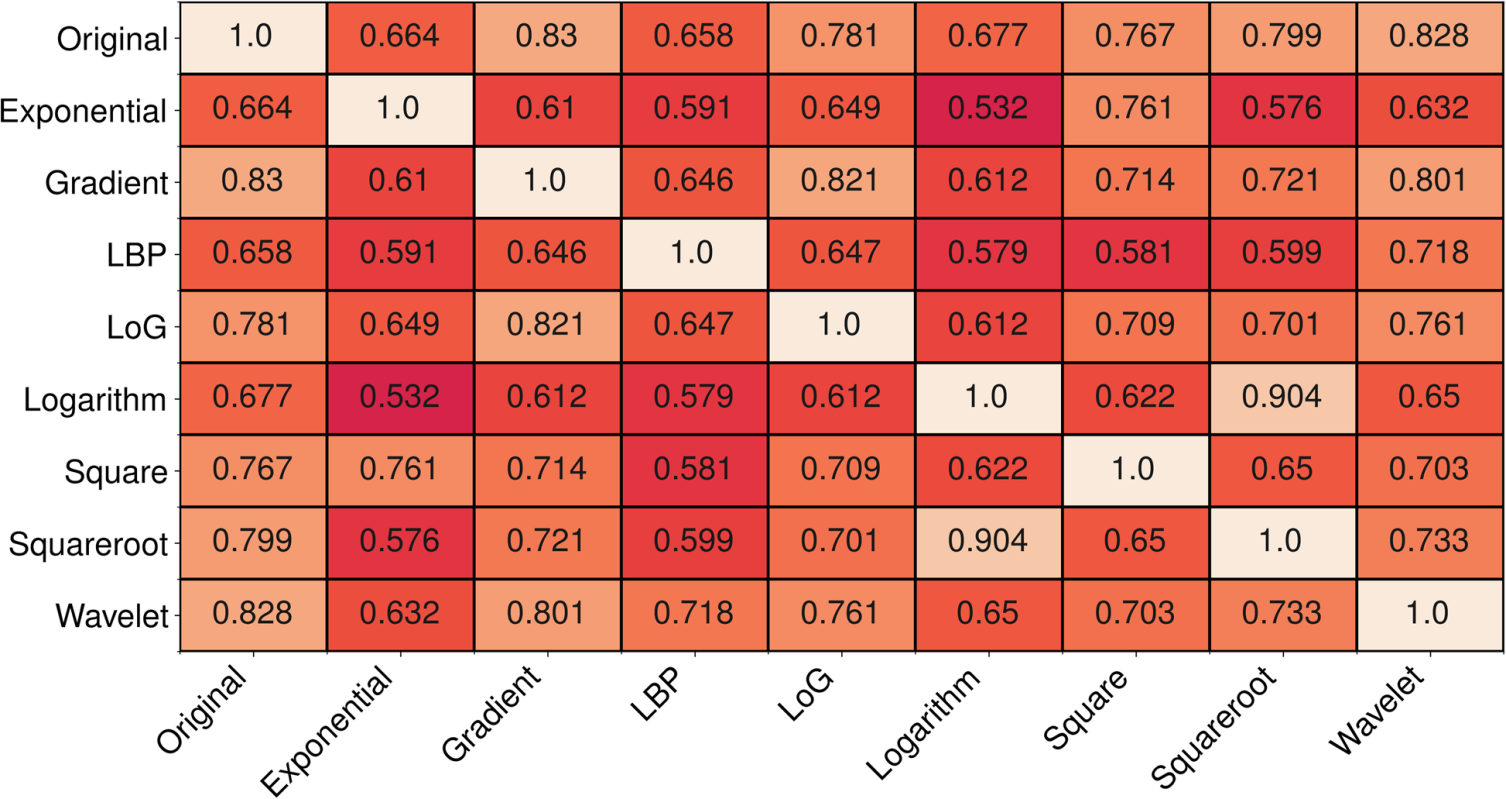
Mean correlation between feature sets. In each cell, the mean pairwise Pearson correlation coefficient (*r*) between features of the corresponding two feature sets is displayed

The mean correlation over all datasets ranged between *r* = 0.49 and *r* =0.919 (Fig. 4). The local binary pattern and exponential filters showed the lowest correlation to the other feature sets (mean *r* = 0.67 for both), while the correlation of the original feature set was the highest (mean *r* = 0.77).

## Discussion

Image preprocessing filters are commonly used in radiomic studies because they are thought to increase overall prediction performance. However, preprocessing filters are not integral to the standard radiomics pipeline. While some studies did not use preprocessing filters [15–17], other studies use them, generating datasets with several thousand features [18–20]. In the absence of a direct comparison, it is unclear whether the use of such filters can increase predictive performance, as adding more features to the dataset while maintaining the same sample size increases the dimensionality of the dataset, making the training very complex. To understand whether preprocessed features increase the predictive performance, we compared different feature sets using seven publicly available datasets.

Our results showed differences in AUC-ROC when the original set of features or the full set of preprocessed features was used. The difference was highly dependent on the dataset used, and in the majority of cases, a better AUC-ROC was obtained with the original set of features. However, from a statistical point of view, these differences were not significant. On the other hand, the differences were always statistically significant in the three data sets where the use of all features increased performance. Thus, using all features instead of the original feature set does not seem to result into a loss of predictive performance, and it is advisable to use all features in radiomic studies.

Tuning the feature set during cross-validation improved the predictive performance slightly further, and no drop in AUC-ROC was seen compared to the original set of features. This was not surprising since the original set and the set of all features were part of the tuning, and indeed, these sets were performing best on three datasets. However, when the best-performing model is compared to the model trained on all features, the differences were not statistically significant except for the melanoma dataset. Therefore, it is reasonable to tune the feature set during cross-validation to obtain the highest AUC-ROCs, if computational power is of no concern.

Considering the tradeoff between sample size and improvement in AUC-ROC, it seems that when only a few samples are available, the performance of the original feature set is slightly better, even though, as observed, not necessarily statistically so. On the other hand, when more samples are available, the modeling can better deal with the higher dimensionality of the full set of features.

The correlation analysis between the different feature sets showed that they are quite strongly correlated and have a pairwise Pearson correlation coefficient of about 0.70. In other words, each feature from one set is correlated with a feature from the other set with an average correlation of *r* = 0.70. However, this should be taken with caution because the correlation only accounts for a linear association, whereas machine learning models can utilise nonlinear associations. Therefore, the high correlation does not directly reveal the performance of the feature set.

Overall, our study shows that the increased dimensionality that results from the application of preprocessing filters does not significantly affect predictive performance. While one could argue that this is not surprising since feature selection methods can potentially remove those features that are not helpful and thus reduce the increasing dimensionality to a manageable level, it is known that feature selection is generally unstable at high dimensions [21, 22]. However, we have shown that the features coming from the preprocessing filters are quite highly correlated with the original set and with each other. This correlation may be the reason why, despite unstable feature selection methods, increased dimensionality does not entail a loss in predictive performance.

The focus of our study was the impact of the preprocessing filters on the predictive performance, which was not systematically studied in previous studies. Most of them were conducted to assess the impact of preprocessing filters on the variability and reproducibility of the extracted features. For example, Rinaldi et al. [5] studied the impact of scanner vendor and voltage on the features extracted from computed tomography (CT) scans in patients with non-small-cell lung cancer (NSCLC). They showed that wavelet features have lower stability than LoG features. Using CT scans from patients with NSCLC, Fave et al. [6] analysed how far applying preprocessing filters changes the volume-dependency of features and their univariate correlation with overall survival. Their results showed that preprocessing filters have a large impact on both. Um et al. [7] studied the effect of preprocessing filters, namely rescaling, bias field correction, histogram standardisation, and isotropic resampling, on the scanner dependency of features extracted from magnetic resonance imaging (MRI) of patients with gliomas. They concluded that histogram standardisation has the largest impact. In the same spirit, Moradmand et al. [8] showed that bias correction and denoising have a large and different effect on the reproducibility of features preprocessed with image filters like wavelet and LoG. These effects were not in the focus of our study, although they are key for clinical application, since preprocessing filters can potentially reduce noise, harmonise feature values coming from different scanners [23], and reduce interobserver variability [24]. In other words, they can have a large effect on the both, reproducibility and generalizability.

In our study, we used PyRadiomics for feature extraction and preprocessing. Strictly speaking, our results, therefore, only apply to this software package. Since other radiomics software packages may use different definitions of the preprocessing filters, it is unclear whether our observations also apply to these software packages. The Image Biomarker Standardization Initiative has recently started a project to arrive at a standardised definition of preprocessing filters to achieve more reproducibility [25].

For the radiomics pipeline we used in this study, we had to make several decisions that could potentially have an impact on the results and on the reproducibility of the models. One is data quantization; in this case, we decided to use a fixed bin width of 25, which is the default and recommended method in PyRadiomics [11]; however, the Image Biomarker Standardization Initiative recommends a fixed number of bins [26]. Ideally, this decision should be considered a hyperparameter and be determined for each dataset individually. However, since a different quantization affects all feature sets equally, we expect our statements for other quantization to be valid as well. Therefore, we repeated the experiments with fixed bin numbers of 100 and observed that the conclusions of our study still hold (the results can be found in the Supplemental material). Another parameter of the radiomics pipeline that might affect the results is the normalisation of the data; here, we have chosen to normalise only the MRI data, but not the CT data, since CT scans are comparable to each other in terms of the intensity values. However, it has been shown that normalisation can be useful even in this case. For example, Schwier et al. [27] demonstrated that normalising apparent diffusion coefficient data leads to a higher reproducibility of the features. There are further decisions that can highly affect the predictive performance of the models, including the validation scheme, choice of feature methods, and classifiers. Thus, for a given dataset, it is reasonable to optimise all these choices to obtain a most predictive and reproducible model; for example, a decorrelation step might be employed to further counteract the negative effects of high dimensionality of the datasets.

Ultimately, the clinical usefulness of radiomics can only be judged using external datasets. Since such datasets with high sample numbers coming from multiple centres are still missing largely, we employed nested cross-validation. Therefore, our results can be seen as preliminary. In addition, not using explicit validation data might give rise to a positive bias, even though nested cross-validation should give a relatively unbiased estimation, if the external data follows the same distribution [13]. Comparing our AUC-ROCs to those of Starmans et al. [28], it is striking that using the original set of features on four datasets (Desmoid, GIST, Lipo, Liver), the performance is slightly below the 95% CI reported there. For using all features, only in one case (Liver) was the performance lower, while no difference could be seen when using the best feature set. This confirms that using too few features indeed might hurt the predictive performance of the models. Nonetheless, one must keep in mind that our pipeline was different from that of Starmans et al. [28]. For example, in our study, all scans were homogeneously resampled since it was shown that this might increase predictive performance [29]. In addition, they only used a slice-based approach to extract texture features which could also affect the results, as our study shows. They also use preprocessed features but only extract 534 features. In addition, their validation scheme is different from ours.

In conclusion, preprocessing filters can have a large influence in radiomic studies. Our results demonstrate that even though they increase the dimensionality of the dataset, they can improve the predictive performance of the models.

## Supplementary Information

**Additional file 1.**

## Data Availability

The datasets analysed during the current study and the evaluation code are available in the github repository, https://github.com/aydindemircioglu/radPreFilter.

## Abbreviations

AUC-ROC: Area-under-the-curve at receiver operating characteristics analysis
CT: Computed tomography
LASSO: Least absolute shrinkage and selection operator
LoG: Laplacian-of-Gaussian
MRI: Magnetic resonance imaging
NSCLC: Non-small-cell lung cancer

## References

[1] Rizzo S, Botta F, Raimondi S (2018). Radiomics: the facts and the challenges of image analysis. Eur Radiol Exp.

[2] Harlow CA, Dwyer SJ, Lodwick G, Rosenfeld A (1976). On radiographic image analysis. Digital picture analysis.

[3] Gonzalez RC, Woods RE (2018). Digital image processing.

[4] Guyon I, Elisseeff A (2003). An introduction to variable and feature selection. J Mach Learn Res.

[5] Rinaldi L, De Angelis SP, Raimondi S (2022). Reproducibility of radiomic features in CT images of NSCLC patients: an integrative analysis on the impact of acquisition and reconstruction parameters. Eur Radiol Exp.

[6] Fave X, Zhang L, Yang J et al (2016) Impact of image preprocessing on the volume dependence and prognostic potential of radiomics features in non-small cell lung cancer. Transl Cancer Res 5. 10.21037/8709

[7] Um H, Tixier F, Bermudez D, Deasy JO, Young RJ, Veeraraghavan H (2019) Impact of image preprocessing on the scanner dependence of multi-parametric MRI radiomic features and covariate shift in multi-institutional glioblastoma datasets. Phys Med Biol 64:165011. 10.1088/1361-6560/ab2f44 10.1088/1361-6560/ab2f44 31272093

[8] Moradmand H, Aghamiri SMR, Ghaderi R (2019). Impact of image preprocessing methods on reproducibility of radiomic features in multimodal magnetic resonance imaging in glioblastoma. J Appl Clin Med Phys.

[9] Starmans MPA, Timbergen MJM, Vos M et al (2021) The WORC database: MRI and CT scans, segmentations, and clinical labels for 930 patients from six radiomics studies. medRxiv:2021.08.19.21262238. 10.1101/2021.08.19.21262238

[10] Aerts HJWL, Velazquez ER, Leijenaar RTH (2014). Decoding tumour phenotype by noninvasive imaging using a quantitative radiomics approach. Nat Commun.

[11] van Griethuysen JJM, Fedorov A, Parmar C (2017). Computational radiomics system to decode the radiographic phenotype. Cancer Res.

[12] Lambin P, Leijenaar RTH, Deist TM (2017). Radiomics: the bridge between medical imaging and personalized medicine. Nat Rev Clin Oncol.

[13] Varma S, Simon R (2006). Bias in error estimation when using cross-validation for model selection. BMC Bioinformatics.

[14] Pedregosa F, Varoquaux G, Gramfort A (2011). Scikit-learn: machine learning in Python. J Mach Learn Res.

[15] Carvalho S, Leijenaar RTH, Troost EGC (2018). 18F-fluorodeoxyglucose positron-emission tomography (FDG-PET)-radiomics of metastatic lymph nodes and primary tumor in non-small cell lung cancer (NSCLC) – a prospective externally validated study. PLoS One.

[16] Wang J, Kato F, Oyama-Manabe N (2015). Identifying triple-negative breast cancer using background parenchymal enhancement heterogeneity on dynamic contrast-enhanced MRI: a pilot radiomics study. PLoS One.

[17] Braman NM, Etesami M, Prasanna P (2017). Intratumoral and peritumoral radiomics for the pretreatment prediction of pathological complete response to neoadjuvant chemotherapy based on breast DCE-MRI. Breast Cancer Res.

[18] Mao B, Zhang L, Ning P (2020). Preoperative prediction for pathological grade of hepatocellular carcinoma via machine learning–based radiomics. Eur Radiol.

[19] Haubold J, Demircioglu A, Gratz M (2019). Non-invasive tumor decoding and phenotyping of cerebral gliomas utilizing multiparametric 18F-FET PET-MRI and MR Fingerprinting. Eur J Nucl Med Mol Imaging.

[20] Wang X-H, Long L-H, Cui Y (2020). MRI-based radiomics model for preoperative prediction of 5-year survival in patients with hepatocellular carcinoma. Br J Cancer.

[21] Guyon I, Hur AB, Gunn S, Dror G, Saul L, Weiss Y, Buttou L (2004). Result analysis of the NIPS 2003 feature selection challenge. Advances in neural information processing systems 17.

[22] Demircioğlu A (2022). Benchmarking feature selection methods in radiomics. Invest Radiol.

[23] Lennartz S, O'Shea A, Parakh A, Persigehl T, Baessler B, Kambadakone A (2022) Robustness of dual-energy CT-derived radiomic features across three different scanner types. Eur Radiol 32:1959–1970. 10.1007/s00330-021-08249-2 10.1007/s00330-021-08249-2 34542695

[24] Gitto S, Cuocolo R, Emili I (2021). Effects of interobserver variability on 2D and 3D CT- and MRI-based texture feature reproducibility of cartilaginous bone tumors. J Digit Imaging.

[25] Depeursinge A, Andrearczyk V, Whybra P (2021). Standardised convolutional filtering for radiomics. arXiv:2006.05470 (eess.IV).

[26] Zwanenburg A, Vallières M, Abdalah MA (2020). The image biomarker standardization initiative: standardized quantitative radiomics for high-throughput image-based phenotyping. Radiology.

[27] Schwier M, van Griethuysen J, Vangel MG (2019). Repeatability of multiparametric prostate MRI radiomics features. Sci Rep.

[28] Starmans MPA, van der Voort SR, Phil T (2021). Reproducible radiomics through automated machine learning validated on twelve clinical applications. arXiv:2108.08618v1 (eess.IV).

[29] Mackin D, Fave X, Zhang L (2017). Harmonizing the pixel size in retrospective computed tomography radiomics studies. PLoS One.

