# Supplementary material for: The effect of preprocessing filters on predictive performance in radiomics
Source: Eur Radiol Exp. 2022 Sep 1;6:40. doi: 10.1186/s41747-022-00294-w (PMC9433552; doi:10.1186/s41747-022-00294-w)

ELECTRONIC SUPPLEMENTARY MATERIAL

The effect of preprocessing filters on predictive performance in radiomics

Full list of extracted features

Common features for all preprocessing filters

firstorder_10Percentile

firstorder_90Percentile

firstorder_Energy

firstorder_Entropy

firstorder_InterquartileRange

firstorder_Kurtosis

firstorder_Maximum

firstorder_Mean

firstorder_MeanAbsoluteDeviation

firstorder_Median

firstorder_Minimum

firstorder_Range

firstorder_RobustMeanAbsoluteDeviation

firstorder_RootMeanSquared

firstorder_Skewness

firstorder_TotalEnergy

firstorder_Uniformity

firstorder_Variance

glcm_Autocorrelation

glcm_ClusterProminence

glcm_ClusterShade

glcm_ClusterTendency

glcm_Contrast

glcm_Correlation

glcm_DifferenceAverage

glcm_DifferenceEntropy

glcm_DifferenceVariance

glcm_Id

glcm_Idm

glcm_Idmn

glcm_Idn

glcm_Imc1

glcm_Imc2

glcm_InverseVariance

glcm_JointAverage

glcm_JointEnergy

glcm_JointEntropy

glcm_MaximumProbability

glcm_SumEntropy

glcm_SumSquares

gldm_DependenceEntropy

gldm_DependenceNonUniformity

gldm_DependenceNonUniformityNormalized

gldm_DependenceVariance

gldm_GrayLevelNonUniformity

gldm_GrayLevelVariance

gldm_HighGrayLevelEmphasis

gldm_LargeDependenceEmphasis

gldm_LargeDependenceHighGrayLevelEmphasis

gldm_LargeDependenceLowGrayLevelEmphasis

gldm_LowGrayLevelEmphasis

gldm_SmallDependenceEmphasis

gldm_SmallDependenceHighGrayLevelEmphasis

gldm_SmallDependenceLowGrayLevelEmphasis

glrlm_GrayLevelNonUniformity

glrlm_GrayLevelNonUniformityNormalized

glrlm_GrayLevelVariance

glrlm_HighGrayLevelRunEmphasis

glrlm_LongRunEmphasis

glrlm_LongRunHighGrayLevelEmphasis

glrlm_LongRunLowGrayLevelEmphasis

glrlm_LowGrayLevelRunEmphasis

glrlm_RunEntropy

glrlm_RunLengthNonUniformity

glrlm_RunLengthNonUniformityNormalized

glrlm_RunPercentage

glrlm_RunVariance

glrlm_ShortRunEmphasis

glrlm_ShortRunHighGrayLevelEmphasis

glrlm_ShortRunLowGrayLevelEmphasis

glszm_GrayLevelNonUniformity

glszm_GrayLevelNonUniformityNormalized

glszm_GrayLevelVariance

glszm_HighGrayLevelZoneEmphasis

glszm_LargeAreaEmphasis

glszm_LargeAreaHighGrayLevelEmphasis

glszm_LargeAreaLowGrayLevelEmphasis

glszm_LowGrayLevelZoneEmphasis

glszm_SizeZoneNonUniformity

glszm_SizeZoneNonUniformityNormalized

glszm_SmallAreaEmphasis

glszm_SmallAreaHighGrayLevelEmphasis

glszm_SmallAreaLowGrayLevelEmphasis

glszm_ZoneEntropy

glszm_ZonePercentage

glszm_ZoneVariance

ngtdm_Busyness

ngtdm_Coarseness

ngtdm_Complexity

ngtdm_Contrast

ngtdm_Strength

Features in Original

shape_Elongation

shape_Flatness

shape_LeastAxisLength

shape_MajorAxisLength

shape_Maximum2DDiameterColumn

shape_Maximum2DDiameterRow

shape_Maximum2DDiameterSlice

shape_Maximum3DDiameter

shape_MeshVolume

shape_MinorAxisLength

shape_Sphericity

shape_SurfaceArea

shape_SurfaceVolumeRatio

shape_VoxelVolume

Results for quantization with a fixed number of bins

Using a fixed number of bins (N = 100) instead of a fixed bin width (N = 25) for quantization, the following results were obtained:

	**AUC-ROC Original**	**AUC-ROC All**	**AUC-ROC Tuned**	**Δ All - Original**	**p All - Original**	**Δ Tuned - Original**	**p Tuned - Original**	**Δ Tuned - All**	**p Tuned - All**	**Best Filter**
**CRLM**	0.53 (0.4-0.66)	0.57 (0.44-0.7)	0.66 (0.53-0.78)	0.04	0.665	0.13	0.06	0.09	0.288	lbp-3D
**Desmoid**	0.73 (0.66-0.81)	0.78 (0.71-0.86)	0.78 (0.71-0.85)	0.05	0.247	0.05	0.224	0	0.865	log-sigma-
**GIST**	0.68 (0.61-0.75)	0.76 (0.69-0.82)	0.76 (0.7-0.82)	0.08	0.01	0.08	0.001	0	0.781	square_
**HN**	0.78 (0.7-0.86)	0.78 (0.7-0.86)	0.85 (0.79-0.92)	0	0.996	0.07	0.028	0.07	0.053	square_
**Lipo**	0.77 (0.68-0.85)	0.82 (0.74-0.9)	0.85 (0.78-0.92)	0.05	0.281	0.08	0.035	0.03	0.504	square_
**Liver**	0.71 (0.63-0.78)	0.67 (0.59-0.75)	0.71 (0.63-0.78)	-0.04	0.366	0	1	0.04	0.366	original_
**Melanoma**	0.54 (0.42-0.67)	0.49 (0.37-0.61)	0.63 (0.51-0.75)	-0.05	0.526	0.09	0.24	0.14	0.088	square_

Although there are some differences visible when comparing with Table 4 (e.g., in AUC-ROC Original for CRLM and Lipo), the main conclusions still hold: In no case the AUC-ROCs of the original feature set are significantly better than those of the set of all features. Also, tuning the preprocessing filter never significantly loses performance.

Similarly, there is no large change in the trade-offs and the correlations:

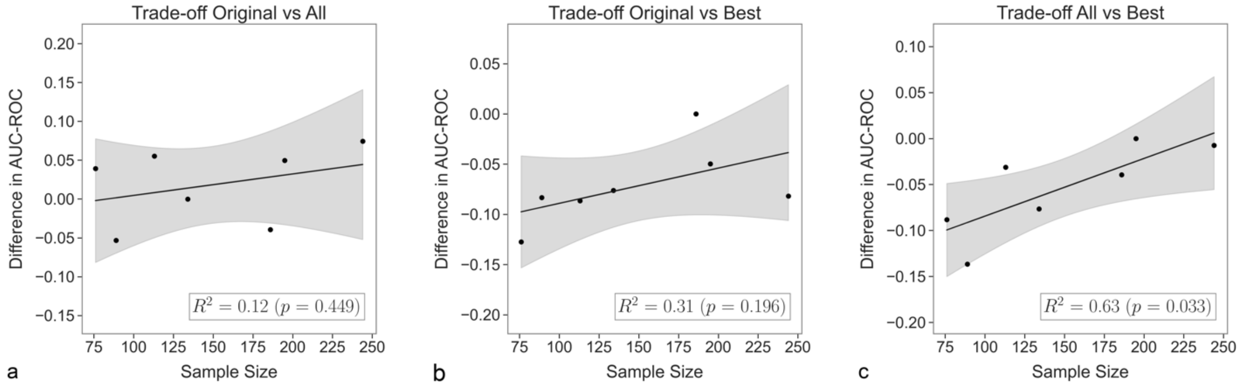

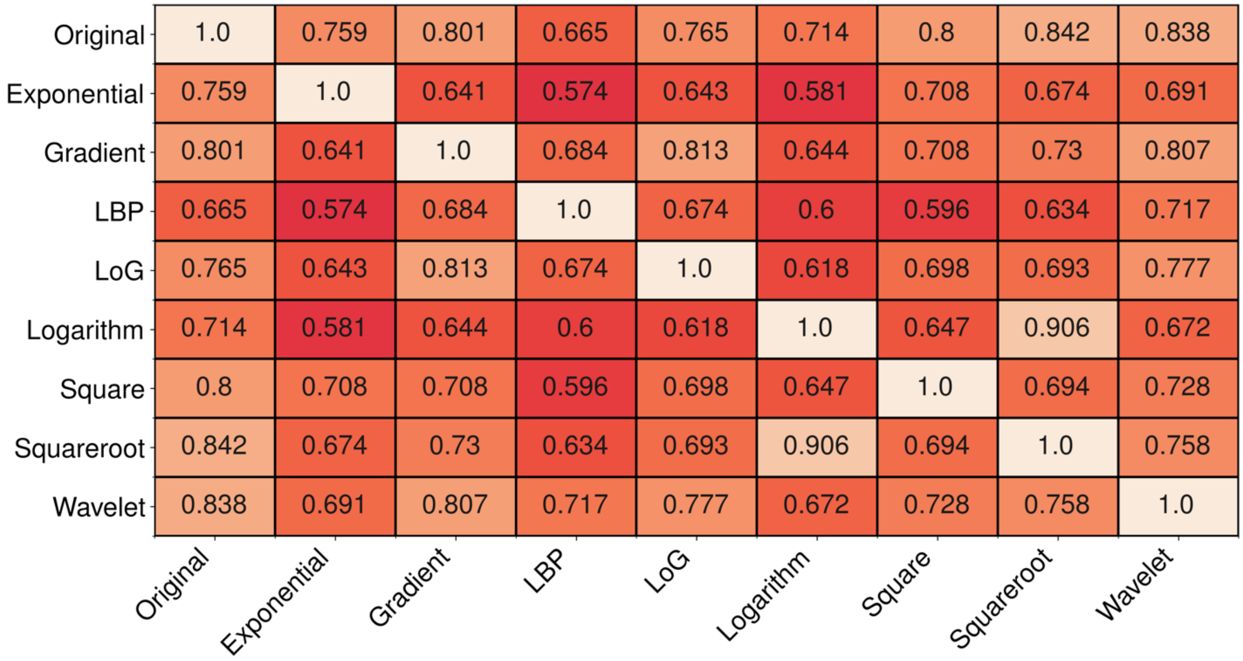

Supplement: Supplementary file 1 — Additional file 1. [file 41747_2022_294_MOESM1_ESM.pdf]
